# A rare case of isolated laryngotracheal amyloidosis with airway narrowing and vocal fold involvement^☆^

**DOI:** 10.1016/j.radcr.2022.07.068

**Published:** 2022-08-27

**Authors:** Mussanna Ahmed, Hamidreza Armani, Navid Salahi, Patrick Hammill

**Affiliations:** a Department of Radiology, SUNY Downstate Health Sciences University/Kings County Hospital, 451 Clarkson Ave, 3rd Fl, Rm B-3304, Brooklyn, NY 11203, USA; b St. George's University School of Medicine, True Blue, Grenada

**Keywords:** Laryngotracheal amyloidosis, Computed tomography, Congo red stain, Apple-green birefringence

## Abstract

Primary amyloidosis is a rare condition with 6-10 cases in a million, with focal involvement representing 9%-15% of those cases [1,2]. Isolated tracheobronchial amyloidosis is extremely rare and when present, can result in focal or diffuse thickening of the glottis, trachea and bronchi, leading to hoarseness, shortness of breath, and dysphonia. Computed tomography (CT) usually shows circumferential thickening of trachea and bronchi with or without calcifications and associated airway narrowing of affected segments. MRI demonstrates intermediate to low signal on T1, low signal on T2 and variable heterogeneous enhancement. Multiple conditions can result in thickening of the airway including but not limited to inflammatory, infectious, and neoplastic etiologies. Biopsy with histologic correlation provides a definitive diagnosis. Biopsied tissue demonstrates characteristic apple-green birefringence with Congo red stain. There is no cure for amyloidosis and the prognosis is quite variable depending on the extent of airway involvement. Current treatments are aimed at alleviating symptoms and include bronchoscopic debridement, laser therapy, and balloon dilation with adjuvant radiation therapy. Here, we present a rare case of a 47-year-old male with isolated laryngotracheal amyloidosis with marked airway narrowing and vocal fold involvement.

## Introduction

Amyloidosis represents a group of disorders characterized by the abnormal deposition of proteins in tissues. There are multiple types of amyloidosis which are broadly characterized as either primary or secondary. Primary amyloidosis or idiopathic amyloidosis is not associated with other diseases and is characterized by abnormal monoclonal light chain immunoglobulins production by plasma cells. Secondary amyloidosis usually occurs in the setting of chronic inflammatory disease or as a result of an immune response. Other types of amyloidosis include but are not limited to transthyretin amyloidosis which is inherited and caused by a mutation in the transthyretin gene and Beta-2 microglobulin amyloidosis which occurs in patients with chronic renal failure who are on dialysis.

Isolated tracheobronchial amyloidosis is a very rare condition characterized by submucosal deposition of AL amyloid which can result in progressive airway narrowing which patients presenting with nonspecific symptoms such as hoarseness, stridor and dysphonia. Prognosis is variable and dependent on the extent of airway involvement. Current therapies are aimed at preserving the airway, alleviating symptoms and minimizing recurrence.

## Case report

**Fig. 1:**
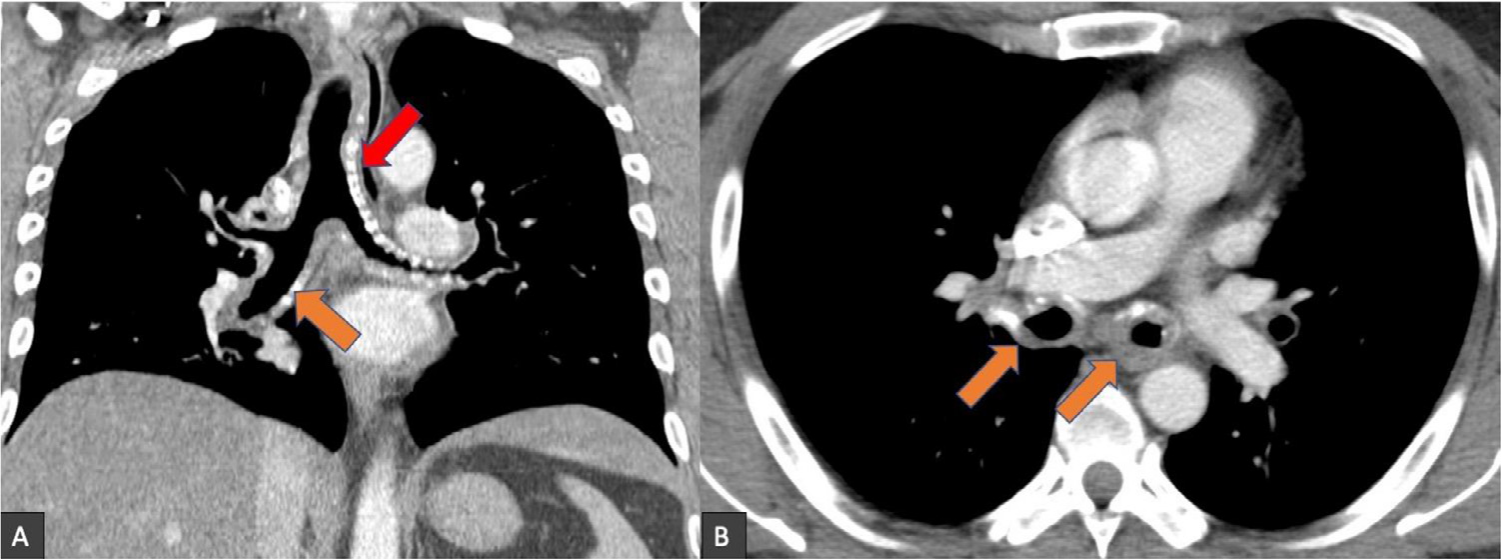
Coronal (A) and axial (B) contrast-enhanced CT of the chest demonstrates marked circumferential soft tissue thickening of the trachea (red arrow) and mainstem bronchi (orange arrows) with coarse calcifications, suggestive of tracheobronchial amyloidosis.

**Fig. 2:**
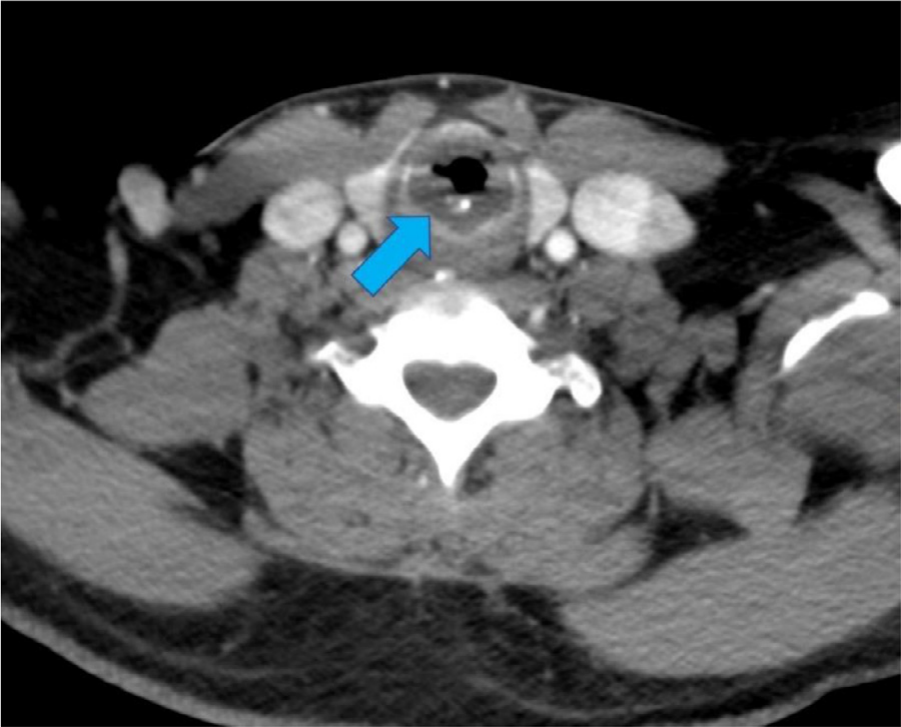
Axial contrast-enhanced CT of the neck with prominent circumferential thickening (blue arrow) of the subglottic airway with calcifications and associated airway narrowing.

**Fig. 3:**
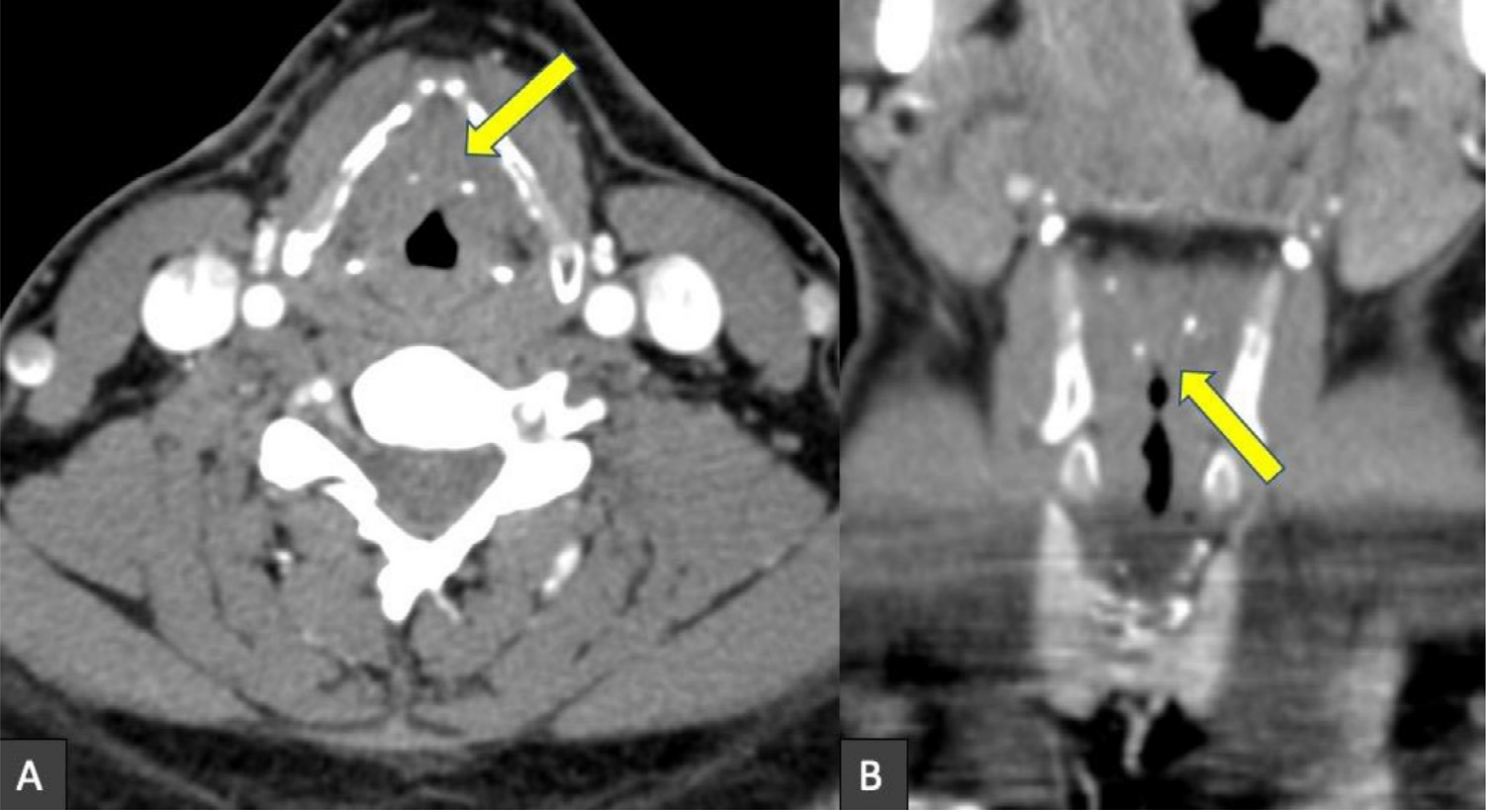
Axial (A) and coronal (B) contrast-enhanced CT of the neck demonstrates nodular thickening of the vocal folds with few coarse calcifications. There is associated airway narrowing, likely resulting in the dysphonia encountered in our patient.

A 47-year-old male presented with chronic progressive worsening shortness of breath on exertion, hoarseness, and dyspnea for several months. Upon admission, the vital signs were stable. Physical exam, basic lab work, and chest x-ray were unremarkable. The patient underwent a contrast-enhanced CT of the neck and chest, which revealed marked circumferential thickening and calcification of the larynx, trachea, and mainstem bronchi with supraglottic, glottic, and subglottic airway narrowing (Figs. 1 and 2). There is involvement of the vocal folds that is manifested as nodularity and calcification (Fig. 3). An extensive workup was conducted to rule out infectious and inflammatory etiologies including QuantiFERON, anti-DNA Ab, anti-nuclear Ab, cyclic citrullinated peptide Ab, Rheumatoid Factor, Hepatitis C Antibodies, and Hepatitis B core and surface antibodies, all of which were negative. Laryngoscopy revealed an inter-arytenoid scar with nodularity and limited abduction of true vocal folds, 60% subglottic region narrowing and thickening of the trachea and mainstem bronchi. A biopsy was performed to rule out malignancy, and subsequent pathologic analysis with Congo red staining and liquid chromatography with tandem mass spectrometry was compatible with primary AL-type lambda amyloidosis. Bone marrow biopsy ruled out any plasma cell neoplasm. Positron emission tomography did not show abnormal hypermetabolic activity. The patient was subsequently diagnosed with localized laryngotracheal amyloidosis arising from monoclonal plasma cells. The patient underwent laryngeal debridement, CO_2_ laser therapy, and balloon dilation with adjuvant radiation therapy, which resulted in marked improvement of the patient's symptoms. However, the patient required multiple follow-up balloon dilations due to disease recurrence.

## Discussion

Primary amyloidosis is a very rare condition with 6-10 cases in a million in the United States. Focal disease involvement represents approximately 9%-15% of those cases [1,2]. Only 1% of primary amyloidosis involves isolated tracheobronchial involvement as seen in this case, with only a few hundred cases reported in the literature [3,4]. Most patients present in the fifth decade of life without definitive gender predilection [5].

Laryngotracheal amyloidosis involves submucosal deposition of AL amyloid and can cause focal or diffuse airway thickening, resulting in marked narrowing of the larynx, trachea, and mainstem bronchi sometimes with vocal cord involvement [2]. Patients commonly present with nonspecific symptoms such as hoarseness, shortness of breath on exertion, dysphonia and wheezing and as such, can be easily misdiagnosed [6]. There are 3 patterns of amyloid airway involvement: proximal, mid, and distal tracheobronchial disease. Patients with the proximal pattern of disease usually present with upper airway symptoms while patients with a mid or distal pattern of involvement can present with lower respiratory symptoms such as recurrent pneumonia or lobar collapse [7]. Differential diagnostic considerations that also can result in diffuse thickening of the upper airway must be considered and excluded including infiltrative neoplasms, sabre-sheath trachea secondary to COPD, granulomatosis with polyangiitis, relapsing polychondritis, sarcoidosis, tracheobronchial tuberculosis, tracheopathia osteochondroplastica, and extra-intestinal manifestations of ulcerative colitis [8].

**Fig. 4:**
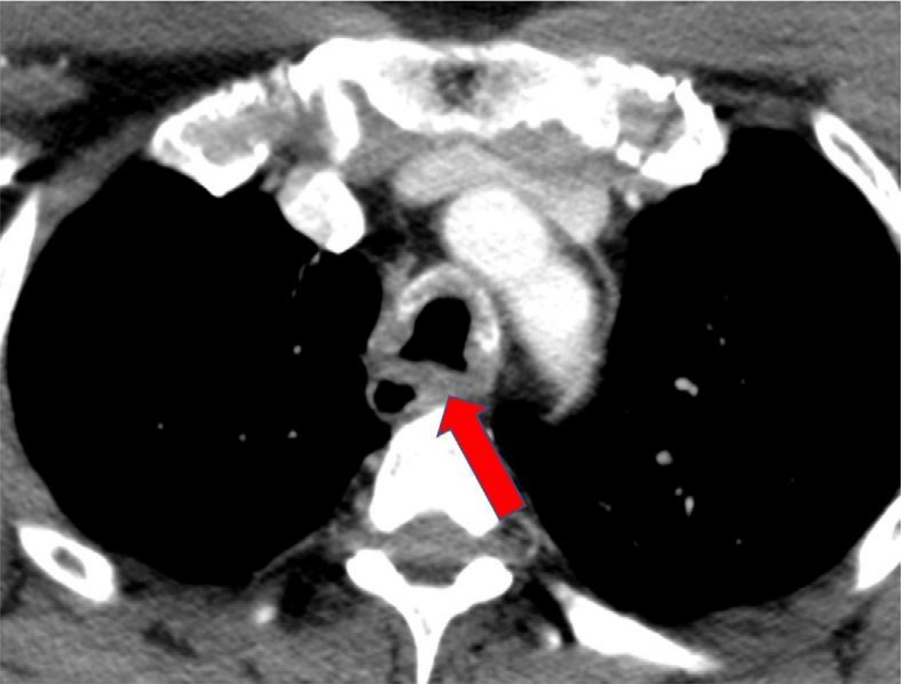
Axial contrast-enhanced CT of the chest at a level above the carina demonstrates circumferential thickening and calcification of the distal trachea without sparing of the posterior wall.

Imaging plays a vital role in the initial evaluation of disease and subsequent response to therapy. Plain film radiographs are nonspecific and can appear normal. Occasionally irregularity and nodularity can be seen in severe cases. CT is the most commonly employed imaging modality and typically demonstrates nodularity, symmetric focal, segmental or diffuse thickening of the airway with resultant airway narrowing. Calcification is frequently seen in longstanding cases. Involvement of the vocal cords is very rare and when present as on this case are seen as thickening and nodularity of the glottic folds. When tracheal involvement is seen, the posterior membrane is not spared (Fig. 4), which is in contradistinction to cartilage diseases such as polychondritis or tracheobronchopathia osteochondroplastica [9]. Direct laryngoscopic or bronchoscopic evaluation is useful for assessment of the vocal cords and glottis and to obtain tissue for biopsy. 18-Fluorodeoxyglucose positron emission tomography when fused with CT may also play a role in early detection and monitoring response to therapy; however, more robust investigation is required [10].

**Fig. 5:**
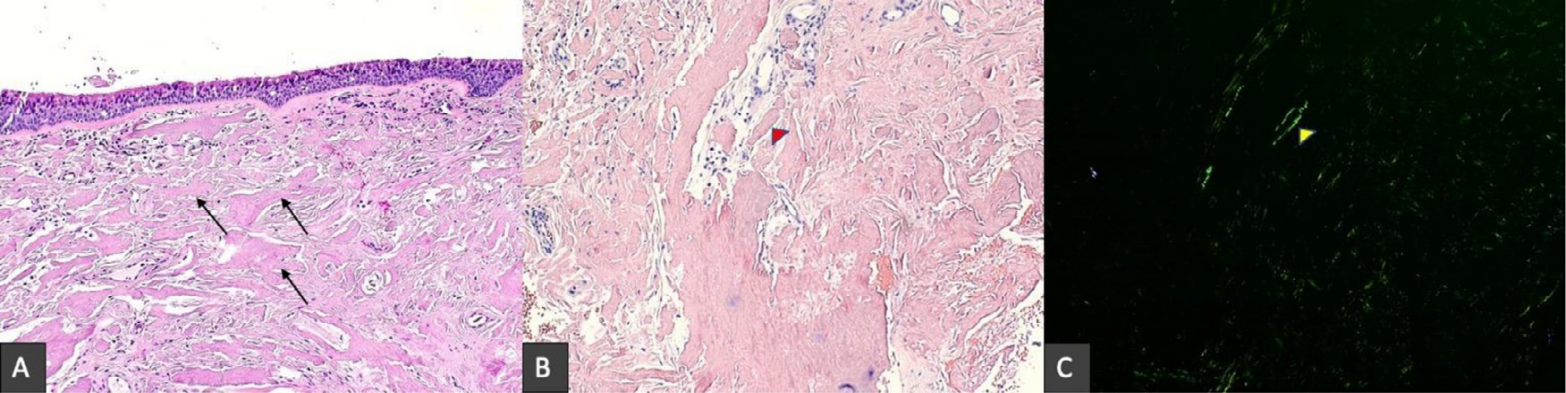
Tracheal biopsy shows a respiratory mucosa with diffuse irregular deposits of eosinophilic material (A, Hematoxylin and eosin (H&E), 100×) with some examples highlighted by the arrows. Diffuse Salmon-colored deposits are seen in Congo red staining (B, Congo red, 200×), with the same slide highlighted with apple-green birefringence under polarized light (C, 200×).The arrowhead is an example of amyloid deposit that is highlighted with apple-green birefringence.

Pathologic analysis of biopsied tissue typically demonstrates abnormally folded extracellular deposits of protein with an affinity for Congo-red stain with characteristic yellow-green birefringence under polarized light (Fig 5) [11]. Amyloidosis can be systemic or localized. Systemic amyloidosis can be further characterized as primary or secondary with primary amyloidosis being secondary to plasma cell dyscrasias and secondary amyloidosis resulting from chronic inflammatory/infectious conditions. Amyloidosis can also be classified biochemically based on the composition of the fibrillar component seen in the protein deposits. To date, over 2 dozen subtypes have been identified with the majority of cases presenting with deposition of amyloid light-chain (AL) and serum amyloid A (AA) [12].

Current therapeutic options include bronchoscopic debridement, laser therapy, and balloon dilation. Disease recurrence has been reported and radiation therapy has shown decreased rates of recurrence [7,8]. Prognosis is generally poor in cases of airway amyloid and is largely determined by the extent of disease and degree of airway narrowing. Severe cases may require tracheostomy or laryngectomy for the preservation of the airway. Complicating factors include airway obstruction and rarely hemorrhage which can be fatal [13], [14]–15].

## Conclusion

We presented a case of a 47-year-old male with isolated laryngotracheobronchial amyloidosis. This condition is very rare and can present with common nonspecific upper airway symptoms. Amyloidosis should be considered as a differential when patients present with hoarseness and shortness of breath, especially when the other common conditions have been ruled out. We summarize the differential diagnostic considerations, characteristic imaging findings and current therapeutic options.
